# An overview of platelet products (PRP, PRGF, PRF, etc.) in the Iranian studies

**DOI:** 10.4155/fsoa-2017-0045

**Published:** 2017-07-28

**Authors:** Seyed Ahmad Raeissadat, Marzieh Babaee, Seyed Mansour Rayegani, Zahra Hashemi, Amir Ali Hamidieh, Parviz Mojgani, Hossein Fouladi Vanda

**Affiliations:** 1 Clinical Research Development Center of Shahid Modarres Hospital & Physical Medicine & Rehabilitation Research Center of Shahid Beheshti University of Medical Sciences, Tehran, Iran; 2 Children's Medical Center, Tehran University of Medical Sciences, Tehran, Iran; 3 Rehabilitation and Medical Education Department, Iran Helal institute of Applied Sciences and Technology, affiliated to the Red Crescent Society of Iran, Tehran, Iran; 4 Rajaie Cardiovascular Medical and Research Center, Iran University of Medical Sciences, Tehran, Iran

**Keywords:** blood platelets, platelet-rich fibrin, platelet-rich growth factor, platelet-rich plasma, PRF, PRGF, PRP, regenerative medicine

## Abstract

**Aim:**

The aim of the study was to carry out a review of published studies on various platelet products in Iranian studies.

**Materials & methods:**

Electronic databases were searched for relevant articles. Two review authors independently extracted data via a tested extraction sheet, and disagreements were resolved by a meeting with a third review author.

**Results:**

Bone disorders (25%), wound and fistula (16%), dental and gingival disorders (14%) and osteoarthritis (11%) have more relative frequency based on different fields.

**Conclusion:**

The necessity of pursuing standard protocols in the preparation of platelet products, stating the precise content of platelets and growth factors, and long-term follow-up of study subjects were the most important points in Iranian studies.

## Lay abstract

Regenerative medicine includes special methods which can repair or replace new tissues beyond relief of symptoms in various diseases. Currently, platelet products (PRP, PRGF, etc.) can be considered as a tool for regenerative medicine. In this study we reviewed Iranian published studies in this field in the last 20 years. The primary object was to provide a clear vision of the quantity and quality of research while reviewing its methods and outcomes. This review highlights the potential application of platelet products, reported complications and their overall efficacy based on Iranian studies, and compares them with recent global findings in these fields.

Regenerative medicine was defined by Mason and Dunnill as the replacement or renewal of the cell, tissues or organs with the aim of restoring their normal function [1]. This relatively new method is considered a primary therapeutic approach, particularly in some chronic illnesses lacking a definite treatment.

Regenerative medicine involves the use of stem cells, growth factor-rich platelets, biologic proteins, gene therapy and so on. Restoration of tissues and the absence of unwanted allograft reactions are among the benefits of this treatment. Platelets are among the cells that have potential in regenerative therapy. Abundance of growth factors and easy availability and processing have given them special attention. Other than growth factors and cytokines (including PDGF, TGF-β, IGF, VEGF) [2], these cells possess anti-inflammatory properties [3] and the cell itself and its products have recently been used in cellular scaffolds [4].

Platelets were first used to activate plasma products (thrombin and fibrinogen) by Cronkite *et al*. in 1944 during skin grafting [5]. Years after, in 1972, Matras *et al*. were the first who presented the idea of using fibrin glue for nerve repair [6,7]. Through the years, use of platelet products for various purposes in animal testing and clinical trials expanded. platelet-rich plasma (PRP) first was described by Marx *et al*. as suspension of platelet in plasma, with higher platelet than original blood collected [8]. Then, a second generation of platelet aggregation (platelet-rich fibrin [PRF]) was introduced which changed the quality of formulation according to speed and time of centrifugation [9,10]. Anticoagulant usage in PRP has been shown to decrease healing. Recently a new product, injectable PRF, was introduced without anticoagulant and different centrifuge speed and time in bone graft [11].

The common point between all studies is lack of definite protocol for preparation of products. Preparations of different platelet products are inconsistent both between and for the same clinician. The various parameters are: venesection (varying quantities and tubes), centrifugation of peripheral blood (varying force, speed and time) and aspiration of a platelet concentrate (varying needle gauge, aspiration technique and size of platelet-rich zone of plasma). It may or may not involve variation in the number of centrifuges (single or double spin), or the use of activation methods (addition of mechanical disturbance, UV light, thrombin or calcium chloride) [12].

In 2009, categorizing platelet products based on leukocyte and fibrinogen content was suggested with four groups [13]: pure PRP (P-PRP), leucocyte- and PRP (L-PRP), P-PR fibrin (P-PRF), and leucocyte- and PR fibrin (L-PRF). Recently, Magalon *et al*. have introduced a new protocol for PRP classification, DEPA classification (dose of injected platelets, Efficacy of production, Purity of the PRP and Activation of PRP) [14].

Along with other countries, in the year 2000, research on the therapeutic applications of platelet derivatives in Iran began with the publication of the results of using autologous PDGFs in the treatment of diabetic ulcers [15]. During the following years, studies have diversified into various areas such as musculoskeletal, skin, wound healing and so on.

With the growing amount of regenerative medicine applications, especially regarding platelets and their derivatives in Iran, we elected to survey the existing published research by Iranian researchers on various platelet products in order to outline their quantitative and qualitative values. We also aimed to assess the current potential in terms of both facilities and personnel such as actively interested centers as well as existing eager individuals in this field. Whatever is gained from this study can be utilized in the improvement of policies for future research and treatment.

## Materials & methods

In this review, the literature search was performed by two people (one specialist and one resident of physical & rehabilitation medicine). Disagreements were resolved by discussion; where resolution was not possible, a third review author was consulted. We included all published studies in Iran that evaluated the various platelet products, irrespective of publication status or language.

The same equivalent Persian words were also used in two valid Persian databases: Iranmedex and Irandoc and relevant articles up to 26 May 2016 were included. We imported the articles obtained from different databases into the bibliographic software package EndNoteX7 and merged them into one complete database.

## Search strategy

In May 2016 we searched the following libraries and electronic databases for potentially relevant studies:

- PubMed
- MEDLINE
- Scopus
- Cochrane central
- Google scholar
- Iranmedex
- Irandoc

The terminologies that were used to identify these articles included:

- # Platelet rich
- # Plasma
- # Growth factor
- # Iran

Furthermore, we used a suitable combination of terminologies as mentioned above for searching.

After removing duplicates, two authors independently assessed the titles and abstracts of all retrieved papers for inclusion, using predefined inclusion and exclusion criteria.

## Inclusion criteria

- Focus of study should be on any of these platelet products: P-PRP, L-PRP, P-PRF, L-PRF and PRGF;
- Study should be done by Iranian researchers and inside Iran;
- Study design should be based on any of these study types: randomized clinical trials, non-randomized clinical trials, case report, systematic review with or without meta-analysis, hypothesis.

## Exclusion criteria

- If focus of study was on platelet disorders and platelet products used through intravascular injection;
- If the study was done by non-Iranian researchers or outside of Iran;
- If the publication type was a letter to editor or conference presentation.

## Data extraction

For the included studies, two authors independently extracted data via a tested extraction sheet, and disagreements were resolved by a meeting with a third review author.

Information was categorized: that regarding platelet disorders and platelet products used through intravascular injection were removed and only articles of any type (except for letters to editor and conference presentation) were included if performed by Iranian researchers and inside the country.

## Results

**Figure 1.**
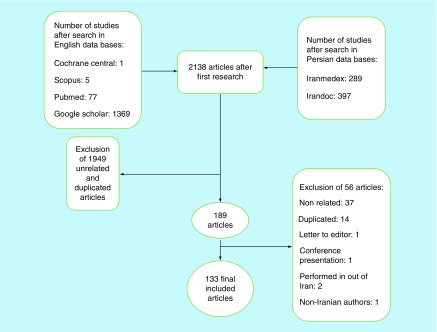
**Stages of search and inclusion and exclusion of eligible articles.**

Among 2138 initial articles, 133 met the desired criteria (Figure 1).

**Figure 2.**
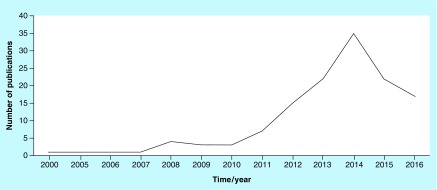
**The number of articles published per year.**

In Iran, the publication of studies related to platelets and their derivatives commenced in the year 2000 and reached its peak in 2014, at which time 35 articles were published regarding this topic (Figure 2).

**Figure 3.**
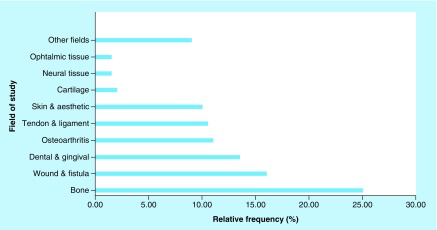
**Relative frequency of studies performed in each field.**

Figure 3 illustrates the relative frequency of studies performed in each of the different fields. The highest numbers belong to bone, wounds, dental and osteoarthritis.

From the different platelet products, PRP (72%), PRGF (13.5%) and PRF (12%) were the most objectives studied.

**Figure 4.**
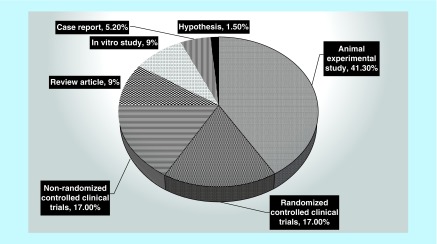
**Relative frequency of each type of study conducted in Iran.**

Animal studies comprised the majority of research, the number of which was 55 (41.3%) and two hypotheses had the lowest number of study types (Figure 4).

Table 1 shows the number of the types of studies performed in different fields. The highest number of human studies has been performed in the dental, osteoarthritis and wound areas.

**Table 1.** **Relative frequency of each type of study in different fields.**

**Study type research field**	**Review articles**	**Randomized clinical trials**	**Non-randomized clinical trials**	**Case reports**	**Animal experimental studies**	***In vitro* studies**
Bone^†^	14.7%	5.8%	11.8%	–	58.9%	5.8%

Wound & fistula	–	22.7%	22.7%	18.2%	36.4%	–

Dental & gingival	–	37.5%	18.7%	6.3%	25%	12.5%

Osteoarthritis	–	26.7%	26.7%	7.1%	39.5%	–

Tendon & ligament	–	21.4%	7.1%	7.1%	64.4%	–

Skin & aesthetic	28.6%	14.3%	28.6%	–	21.4%	7.1%

Cartilage	33.3%	–	–	–	33.3%	33.3%

Neural tissue	–	–	–	–	100%	–

Ophthalmic tissue	–	–	–	–	100%	–

Others field^†^	9%	–	9%	9%	9%	55%

^⊃^Hypothesis of these fields were not included in this table.

One hundred and twenty-five of the 133 studies have been performed in university centers, among which Mashhad university of medical sciences (15.8%), Islamic Azad university (13.5%) and Shahid Beheshti university of medical sciences (12.8%) have the highest number of published papers.

We will now go into reviewing the studies in each area.

## Bone disorders

The highest number of studies regarding platelet products in Iran has been performed in the field of bone disorders (25%). The first of these were performed in 2005 [16] and 2006 [17] on cranial defects in animals. These studies (70% of the total bone studies) cover areas such as repair of various bone defects (including cleft palate [18], site of a limb lengthening osteotomy [19], healing of fractures [20] particularly the non-healing types, osteonecrosis [21] and osteomyelitis [22]).

A variety of platelet derivatives (PRP, PRGF, PPP [23], PRF [24], platelet gel [25] and rh-PDGF [26]) were used in different techniques by Iranian researchers. Concurrent utilization of platelet products and autograft bone [27] or mesenchymal stem cells [24,26,28] in experimental studies as well as evaluation of the growth and differentiation of mesenchymal stem cells [23] for osteogenesis are among them.

Seventy-six percent of the experimental studies have reported good outcomes from the application of platelet products in their studies. All of those studies with no significant findings involved bone defect repair, mostly using PDGFs [26,29–31].

## Wounds & fistulas

Sixteen percent of the studies belong to this area. The first article published regarding platelet products was in the year 2000 by Aminian *et al* [15]. Regarding the use of PDGF on the treatment of diabetic foot ulcers.

The effects of platelet derivatives such as PRP, PRF [32,33] and PGRF [34] were studied on incisional (57%) and diabetic (24%) ulcers, anal fissure (5%) and various fistules (14%).

In 24% of these studies (one regarding anal fistulas and the others concerning incisional wounds), the effectiveness of these modalities was not significant [35–37].

## Dental & gingival disorders

2007 was the first year in Iran that PRP was used by Shamaei *et al*. to treat mandibular molar furcation [38]. After that in 2008, Aminabadi *et al*. suggested the use of PRP for the treatment of recurrent aphthous stomatitis [39]. Pulp capping [40], orthodontics [41], implants [42–44], filling bone defects [45–47], alveolar osteitis [48,49], and hard and soft palate repair [50–52] were afterward studied upon.

## Osteoarthritis

This field includes 11% of all platelet product studies, 53% of which have been in the form of experimental human studies equally divided into RCTs and non-RCTs [53,54].

The study by Kalbkhani *et al*. on animal models [55] and Raeissadat *et al*. on the human knee [56] in 2013 were the first published papers in this field.

PRP injection with or without sport education and its comparison with therapeutic exercise and paracetamol [57], with transcutaneous electrical nerve stimulation and exercise [58], three separate intra-articular injections of Hyalgan [59] and intra-articular steroid injection [60] in knee arthritis are all among the work of Iranian researchers. In all of these studies the effectiveness of PRP was shown to be more than the groups they were compared with. In all the animal studies in this field, platelet products were effective for up to 6 months of follow-up.

## Tendon & ligament injuries

In this field, the effect of platelet derivatives on rupture of anterior cruciate ligament (ACL) [61], lateral epicondylitis [62], plantar fasciitis [63] and frozen shoulder (adhesive capsulitis) [64] comprised the topics studied by Iranians. Among the 14 articles in this area, 9 were performed on animal subjects and 5 on humans (3 RCTs, 1 non-RCT and 1 case report). The first of the studies were performed and published in 2013 concerning the effect of PRP on healing of tendon damage in animal models [65] as well as ACL in humans.

## Skin & aesthetic

By stimulating the fibroblasts [66] and rejuvenation of the skin [67], application of PRP for skin care and aesthetic purposes has been one of the popular fields for Iranian researchers. This field is formed by 10% of all platelet-centered studies and covers treatment of conditions such as wrinkles [68], alopecia [69], grafts [70] and dark circles under the eyes [68]. Review articles and non-RCTs comprise the majority of these studies.

The study of Safari *et al* [71] could be one of the first on skin treatment, which involves assessment of the proliferation of keratinocytes *in vitro* in the presence of PDGF.

## Cartilage grafts

There are a total of three studies in this area. Two of them are on animals and the third is a review article. The first of these was performed in 2012 by grafting a rabbit's auricular cartilage together with PRP application. The viability of the graft increased in this method [72].

## Peripheral nerve damage

The two studies in this area were performed in 2015 [73] and 2016 [74], aiming to repair the sciatic nerve after cutting it in animal models. This has enjoyed better effects regarding the amount of myelin concentration.

## Ophthalmic tissue

Corneal ulcers were the only subject studied in this area, in which two animal studies in 2012 [75] and 2013 [76] were conducted; PRP was found effective.

## Other areas

Reduction in hepatotoxicity in animal studies after PRP injection [77], submucosal and periurethral injection of stem cells derived from autologous peripheral blood combined with PRP for treatment of urinary stress incontinence [78], as well as combination of PRP and fat injection in treating velopharyngeal insufficiency [79] cannot be included in the other categories. Therefore these, along with a variety of *in vitro* studies concerning diverse properties of platelet products, were put into a different category.

Other studies include proof of better results using PRP in fibrin scaffolds for proliferation and sustenance of mesenchymal stem cells compared with fetal bovine serum [80], increased differentiation of stem cells to megakaryocyte precursors in the presence of platelet growth factors [81], comparison of the mechanical properties of the membrane resulting from Early L-PRF compared with PRGF-Endoret system [82], proposal of a new method for platelet gel preparation using a special mixture of PRP, thrombin and calcium [83], and demonstrating that growth factors and platelet gel produced from expired platelets have the same concentration and function as those from fresh platelets [84].

## Complications of PRP

Due to autologous injection, there is no immune reaction or allergy. Indeed, severe side effects are rare in the literature. The main risks are pain and infection. Use of a pain killer (non-NSAID) or local anesthesia decrease the pain. Another prevalent complication is heaviness in the site of injection [57,59].

## Discussion

### Bone disorders

Autogenous bone grafts are the gold standard for craniofacial and orbital bone defect repair [85]. Because of limitations in access as well as problems at the donor site (such as chronic pain and infection), tissue engineering using cell therapy (mesenchymal stem cells) [31], growth factors (PRP, PRGF) [86–88] and a combination of the both [89–92] has been suggested as bone graft alternatives in recent decades [26,93]. Iranian researchers have conducted human [94] and animal studies using different platelet products as well as their combination with various substances such as human amniotic fluid [95], hydroxyapatite [96,97], Persian Gulf coral [98] and deproteinized bovine bone mineral [99] for bone regeneration.

According to the review articles published in 2014 [93], 2015 [89] and 2016 [100], because of the differences in PRP preparation methods as well as the low number of controlled clinical studies, sufficient clinical evidence is lacking. Therefore, despite all of the merits associated with PRP, it should not yet be considered as a first-line therapy in this area. A review article on PRP applications in non-union of traumatic fractures in 2007 arrived to the same conclusion [101].

In 2007, Namazi pointed out the possibility of PRP use in osteomyelitis treatment, due to the presence of angiogenesis stimulating factors (such as VEGF) [22]. A number of human and animal studies showed its effectiveness in treatment of osteomyelitis of various bones [102–104].

### Wounds & fistulas

The majority of Iranian studies have performed RCTs on: incisional wounds [105–107], 42% of which have been performed on humans, including postcesarean section [108], pilonidal abscess extraction [109]. The non-RCTs include sternotomy [110] and soft tissue sarcoma excision [111] as a case report. The only study not demonstrating a meaningful effect on wound healing was the pilonidal sinus surgery. All studies conducted on diabetic foot ulcers [112–114] and fistulas [115–117] confirm the effect. An analysis of ten RCTs covering chronic ulcers, diabetic foot ulcers and venous ulcers was performed by Cochrane in 2016 [118]. Because of the small, low-quality RCTs in this study, the effectiveness of PRP on diabetic foot ulcers can only be considered as probable; and because of the low strength of the studies, the treatment effect was not possible to calculate.

Use of modern modalities such as PRP in conjunction with Chitosan Biodegradable Film in animal studies is also among the research related to wound healing performed in Iran [119].

### Dental & gingival problems

The effect of platelet derivatives on wound healing has led to the idea of their use in various dental and gum disorders. In the comparison between PRGF and PRF *in vitro*, PRGF showed a higher stimulatory role in the survival and proliferation of human gingival fibroblasts [120] and was considered as a means of periodontal regeneration.

Platelet derivatives also have an important role in plastic surgery as preimplant connective tissue grafts, in healing of the graft donor site and prevention of adverse effects. In 2010, PRP was used in the donor site of animal models, although a meaningful effect was not seen regarding healing acceleration [121].

In addition, PRP effects have been studied on the proliferation and differentiation of pulp stem cells of humans [122].

### Osteoarthritis

In a study, the prevalence of osteoarthritis in at least one joint in Iran was reported to be 20%, 19.34% of which affect the knee [123]. Therefore, knee osteoarthritis is among the most popular fields for Iranian researchers.

Animal studies commonly employ surgically induced osteoarthritis [124–126] followed by evaluating the effect of platelet products such as PRP, L-PRP [127] or PRF [128] in joints.

Based on a systematic review and meta-analysis conducted by Iranian researchers in 2016, the effectiveness of injection of hyaluronic acid and PRP in the knee joint was compared among 722 patients (from seven studies). Based on the WOMAC questionnaire, although both modalities were effective, PRP showed more promising outcomes [129]. The meta-analysis performed by Campbell *et al*. in 2015 [130] also pointed out the larger effect size, up to 1 year, of PRP injection compared with hyaluronic acid on the function of the knee. It seems that moderate-quality evidence supports the use of platelet in knee osteoarthritis [131].

In another study, PRP was compared with physiotherapy in treatment of chondromalacia [132]. A main drawback of this study was not restricting the use of NSAIDs until 1 week after intervention in both groups, since NSAIDs after treatment could significantly reduce the effects of PRP [131].

### Tendons & ligaments

Tendon injuries have always been a challenge for patient rehabilitation, in particular sports injuries. In most human and animal studies fitting within the scope of this article, PRP has been reported as effective [65,133]. Moderate-quality evidence shows the effectiveness of PRP injection in chronic lateral epicondylitis but its superior effect compared with autologous whole blood in treatment of this problem [62,134] and plantar fasciitis [63] was not shown. Corticosteroid injection is another optional treatment for them [135].

The studies regarding Achilles tendon repair have all been performed on animals and PRP injection alone or in conjunction with laser therapy [136,137], hydrotherapy [138]. In some of these studies PRP injection has shown a meaningful effect [139–141], while in others the effect has not been significant [142].

### Skin & aesthetic

Because of the increase in the lifetime of adipose cells and stem cells together with higher differentiation, a mixture of PRP and autologous fat cells has been used as a deep filler for nasolabial, perioral and cheek wrinkles [143–145].

In a laboratory experimentation, Ahmadi-Ashtiani *et al*. [146] discovered that the combination of PRP and an herbal extract has induced increased proliferation and lifetime of papillary derm cells, which could herald hopes for a treatment of hair loss. Bagherani's review article [69] also evaluated and approved PRP as a safe and effective treatment of alopecia areata although, in a review article, Jaffari [147] considered the level of evidence for the effectiveness of PRP in hair loss and hair grafting to be low to intermediate and the number of existing articles to be limited. According to her study, although current evidence confirms PRP's role in hair grafting, more research with higher level evidence is still required.

Other work used a type of laser in combination with simultaneous PRP injection for the treatment of acne, which not only did show a synergistic effect, but the results were worse [148].

In 2016, PRP and PRF were compared with a control group regarding the survival of a full skin graft in animals. After 4 weeks, PRP showed no meaningful difference with the controls and the results of PRF were actually worse than controls [70].

### Cartilage

Since cartilage is an avascular tissue with little capacity for self-repair, tissue engineering via mesenchymal stem cells, cellular scaffolds and chondrogenic growth factors are of great significance.

In their review article, Kabiri and colleagues [149] confirmed the positive influence of PRP in chondrogenesis. They believe the various methods of PRP preparation in different studies as well as failure to mention the concentration of growth factors in the products to be among reasons for the contradictory results seen in some studies.

### Peripheral nerves

In 2015, a silicon tube filled with PRP [73], and in 2016 PRP injection under the perineurium proximal and distal to a cut in the sciatic nerve [74] were compared with a control group (only normal saline) in animals. There was no significant difference in the number of axons.

By taking a glance at the studies performed, the diversity of platelet products as well as their various uses can be observed. The range of studies of Iranian scientists shows the high potential presence of different products with various goals available to the patients. To the same extent, the heterogeneous nature of the study results makes judgement and clinical decision-making a challenge in this field.

According to studies, the growth factor concentration, platelet count, presence of other cells such as leukocytes as well as the mechanism of platelet content release all vary based on the method which the PRP has been prepared [150,151]. Therefore, following standard protocols and mentioning the aforementioned properties of the prepared products seems necessary, which is lacking in some studies. Creating and committing to standard principles in preparation of platelet derivatives is one of the requirements of their presence in clinical practice.

Based on a review article by Moshiri, although more than 90% of the *in vivo* and *in vitro* studies acknowledge the effectiveness of PRP in tissue regeneration, more than 50% of human studies have not reached positive findings. Researchers believe that in most animal studies, allogenic PRP is used, while in human studies the PRP is mainly autologous. They have come up with the hypothesis that because of the inflammatory process caused by allogenic PRP injection, acceleration of the tissue regeneration process is more possible [152].

While it has been mentioned that PRP can affect the growth of tumors [153], there is no study showing PRP to be cancerogenic due to the presence of growth factors. Long-term safety of these products should also be established through follow-up.

Between the 133 studies, in 1 RCT studying wound healing after pilonidal sinus removal, there was a higher rate of infection in the PRP group compared with the control group [109], which has not been reported in other similar studies. This makes description of the exact method of preparation and injection as well as a comparison between presence of coexisting diseases in control and intervention groups imperative prior to any conclusions regarding side effects of PRP injection.

Another important consideration is proper outcome assessment. Some studies with similar methodologies have sometimes had opposite conclusions. The inclusion of explanations regarding the reasoning behind the conclusion can help the readers and affect the final conclusion. Age group of those studied as well as the duration of follow-up of effects should also be included in study evaluation [154].

### Rules of PRP use in Iran

During the past few years, and along with the results of scientific literature, the ministry of health and medical education has gradually changed their stance regarding PRP. In 2011, clinical workshops and provision of continuous medical education certificates were banned. Two years later, manufacture and sale of the first PRP preparation kit was approved for orthopedic uses only, and trained physicians allowed to use the kits for musculoskeletal disorders.

In 2015, the Iranian Food and Drug Organization approved the use of PRP in treatment of chronic ulcers, regeneration of musculoskeletal soft tissue, and pain reduction in knee osteoarthritis (when steroids and hyaluronic acid have shown to have no effect, and as pain relief until joint replacement). It has also been stated that due to evidence on skin rejuvenation not being sufficient, PRP use in cosmetic procedures has not yet been approved by this association.

To compare this with other countries, the USA presented a paper about ‘US Definitions, Current Use and FDA Stance on Use of Platelet-Rich Plasma in Sports Medicine’. In this article, PRP falls under the purview of the US FDA's Center for Biologics Evaluation and Research. According to the code of regulation (FDA's 21 CFR 1271), it is not necessary for blood product to follow the FDA's traditional regulatory pathway that includes animal studies and clinical trial. For bringing PRP preparation systems to the market, the 510(k) application is recommended. Today most of these products are related to use and a mix of PRP for bone graft [155]. Two other studies investigated the effect of PRP in musculoskeletal disorders in Korea and Australia [12,156]. The Australian paper studied the pattern of PRP among sport physicians and the Korean paper discussed PRP therapy in knee joint problem and legal perspective in Korea.

As far as we know, there is no similar literature in other countries which survey all fields related to platelet products. However, there is an increasing trend in application of platelet products in other countries especially in developing countries such as Turkey, Egypt, Saudi Arabia and so on. According to our research in the Medline/PubMed database regarding PRP application in the aforementioned countries, Turkish researchers and researchers of Saudi Arabia have the most and least publications in this area, respectively. In Turkish studies, just like Iranian research, effects on bone and joints comprised the majority of the work. In Turkey, dental and tendon fields had the second highest amount of research, but in our studies wound healing is the second most common. Based on our knowledge, Turkish researchers do not have any publications on ophthalmic disorders but they have performed several studies concerning the ENT field and PRP protocols. Egyptians had fewer publications than Iranians in the application of PRP in bone and joint disorders, and skin and aesthetics have been the most popular fields for them. Generally, these differences in numbers of published studies do not necessarily reflect the interest of application of platelet product in their countries and may have been affected by more common health problems and accessibility to the platelet products as well as the governmental rules for use of these products.

## Conclusion & future perspective

Despite the lack of statistics about scope of studies in other countries regarding use of platelet derivatives, it seems that Iranian researches follow this topic seriously, particularly in academic centers. Gradually the quality of research is improving, and the fields of platelet-product-studies are expanding which could lay the foundation of further, and at the same time more logical, clinical applications.

The necessity of pursuing standard protocols in preparation of the platelet products, stating the precise content of platelets and growth factors and long-term follow-up of study subjects were the most important factors for Iranian studies.

**Executive summary**

- There is growing trend toward regenerative medicine for management of chronic and disabling diseases.
- Platelet cells play a key role in recent research owing to effects of their various growth factors and cytokines with healing properties.
- PRP with 72%, then PRGF (13.5%) and PRF (12%) were the most attractive platelet products in Iranian research.
- 41.3% of studies utilized an animal experimental method, while randomized clinical trials (17%) and non-randomized clinical trials (17%) were the next in rank.
- Bone disorders (25%), wound and fistula (16%), dental and gingival disorders (14%) and osteoarthritis (11%) have more relative frequency based on different fields.
- The necessity of pursuing standard protocols in the preparation of the platelet products, stating the precise content of platelets and growth factors and long-term follow-up of study subjects were the most important factors in Iranian studies.
